# Demographic and clinical trends of young breast cancer patients from the national cancer database: disproportionate effect on minority populations

**DOI:** 10.1007/s10549-024-07588-0

**Published:** 2025-01-05

**Authors:** Cynthia Mark, Vivek Pujara, Marissa K. Boyle, Yuan Yuan, Jin Sun Lee

**Affiliations:** 1 Division of Medical Oncology, Department of Medicine, Samuel Oschin Comprehensive Cancer Institute, 8700 Beverly Blvd., Los Angeles, CA 90048 USA; 2 Division of Surgical Oncology, Department of Surgery, Cedars-Sinai Medical Center, Los Angeles, CA 90048 USA

**Keywords:** Young breast cancer, Underrepresented populations

## Abstract

**Purpose:**

There is an increasing incidence of young breast cancer (YBC) patients with uncertainty surrounding the factors and patterns that are contributing.

**Methods:**

We obtained characteristics and survival data from 206,156 YBC patients (≤ 40 years of age) diagnosed between 2005 and 2019 from the National Cancer Database (NCDB). Patients were subdivided into two comparison groups based on year of diagnosis (2005–2009, Old vs. 2015–2019, New group). A Chi-square test of independence was employed to measure the changes. Cox proportional hazards model was used to explore the variables influencing overall survival (OS).

**Results:**

Comparison between Old (55,397 patients) and New groups (67,930 patients) showed an increase in the proportion of Hispanic (8.4% vs 10.0%), Black (16.0% vs 16.6%), and Asian (4.8% vs 6.7%) populations. In the New group, black patients had a significantly worse OS, p < 0.001. Additionally, there was a reduction in the proportion of patients with private insurance (43,940 (81.7%) vs 51,104 (76.4%)) and an increase in patients with Medicaid (5,893 (11.0%) vs 10,694 (16.0%)). Finally, there was a significant increase in hormone positive disease (35,142 (70.0%) vs 49,409 (75.8%), p = < 0.001).

**Conclusions:**

Within YBC patients, the proportion of underrepresented and underserved population is increasing, with an impact on OS. We also see an increase in hormone positive disease. Awareness of these at-risk populations is important for early identification of breast cancer and mitigation of poorer outcomes. Also, there are increasing rates of hormone positive disease which can cause substantial personal and societal implications, such as impacts on family planning and early menopause.

## Introduction

Breast cancer is the most common malignant tumor in women. Over the past decades, we have seen the incident cases of breast cancer increase from 876,990 in 1990 to 2,002,350 in 2019 [1]. According to the American Cancer Society, the overall 5-year survival rate for breast cancer patients is 91% but varies widely based on stage of diagnosis as patients with distant spread have an average 5-year survival rate of 31% [2].

One population that has become of increasing interest is patients diagnosed with breast cancer in their premenopausal years. Recent publications have shown increasing incidences in young patients diagnosed with cancer, including breast cancer [3]. While the exact definition of young breast cancer (YBC) can vary from study to study, it is typically defined by women who are diagnosed before the age of 40 [4, 5]. These women are considered to have early onset disease. It is estimated that approximately 10,000 women under the age of 40 are diagnosed with breast cancer in the USA each year [5]. Similarly to the overall incidence of breast cancer, the incident cases of YBC is also increasing. This age group is of particular interest because these patients are diagnosed prior to the age of recommended routine mammographic screening and often have unique tumor biology.

One of the best tools we have for detection and early diagnosis of breast cancer is mammography. Guidelines have varied regarding the typical age of screening. The American Cancer Society recommends yearly mammograms for women ages 45 to 54, with women 55 and older continuing with every other year screening [6]. Previously, the United States Preventative Service Task Force (USPSTF) recommended that women age “50 to 74 years of age who are of average risk for breast cancer get a mammogram every 2 years” [7]. Both guidelines suggested that starting at the age of 40 women should discuss with their doctors or have the choice to begin annual screening. Newer data have shown mortality reduction with annual screening of patients aged 40 to 79 years old as compared to biennial screening of ages 50–74 years old [8]. Data like this led the USPSTF to release a draft recommendation in May of 2023, which was just recently changed to a formal recommendation in April 2024 that women ages 40 to 74 years old should begin every other year screening mammography [7].

Multiple studies have shown that YBC tumors have a unique biology. These tumors tend to be more aggressive when compared with tumors of older women [5]. A greater proportion of these tumors tend to be triple-negative or Human Epidermal Growth Factor Receptor 2 (HER-2) overexpressing tumors [9]. Additionally, given this is typically not a population that is undergoing routine mammographic screening, patients tend to present with a more advanced stage of disease [10]. Given these differences, there tends to be a discrepancy in mortality, with YBC patients having a disproportionate number of lives lost each year [9, 11]. In fact, studies have shown that young age at the time of diagnosis is an independent prognostic factor and is highly associated with increased risk of recurrence and death [12–14].

There have been several studies looking into possible risk factors for developing YBC. Some studies have shown that birth rate, growth rate in childhood, and attained height are all risk factors, but have small effect sizes [5]. A number of studies have looked at diet, exercise, and BMI, but have not found a significant correlation between these factors and development of YBC [15–17]. Even with all the studies evaluating YBC patients, there is still uncertainty surrounding the factors and patterns contributing to the rising incidence in young age. Our study is a retrospective study of the National Cancer Database (NCDB), which aims to evaluate significant changes of demographics and clinical characteristics in YBC patients and how these characteristics can affect overall survival of these patients.

## Materials and methods

We obtained demographic and clinical characteristics as well as survival data of YBC diagnosed with breast cancer between 2005 and 2019 in the National Cancer Database (NCDB). The NCDB is a clinical oncology database from over 1500 Commission on Cancer-accredited facilities. It captures about 70% of newly diagnosed U.S. cancer cases and collects data on demographics, tumor characteristics, and first treatment courses. Patients with YBC were defined as patients diagnosed with breast cancer at or before the age of 40. Patients in the NCDB who were diagnosed under the age of 18 were excluded from the study. Additionally, there are several areas that are reported as “unknown”, and these data points were removed from the analysis.

Data points for some of the categories were captured as different variables in the NCDB PUF dataset depending on the timeframe of record, based on Diagnosis Years Available. Values for our reporting categories were combined as applicable to complete the dataset for our period of analysis. For example, for hormone receptor (HR) subtype determination, estrogen receptor (ER) status was assessed from the Collaborative Stage Data Collection System Site Specific Factor 1 (CS SSF 1) variable prior to 2018, then from the Estrogen Receptor Site Specific Data Item (SSDI) Item 3826 variable 2018 onward. HER-2 status was not collected until the year of 2018, so HER-2 status was not used for the analysis. Combined categories include subtype receptors, clinical T stage, clinical N stage, and clinical stage group. Details of the variables are noted in the NCDB PUF Data Structure and documentation [18].

To evaluate changing demographic and clinical data, the patients were further subdivided into two comparison groups based on year of diagnosis with 5-year intervals (01/2005–12/2009, Old vs. 01/2015–12/2019, New group). Clinical and demographic data between the two groups was compared using a Chi square test of independence, with significance set at a p value less than 0.05.

Further statistical analysis was completed to compare overall survival (OS) based on various demographic or clinical features of each patient. Statistical analysis for determining covariate p-value and hazard ratio was conducted via multivariable Cox proportional hazards regression model using the R survival library ‘coxph’ function. The exponentiated coefficient represents the hazard ratio for each covariate, with a hazard ratio > 1 indicating increased risk of death event for a one-unit increase in the covariate, listed alongside the 95% confidence interval. P-value was associated with the Wald Z-statistic. A p-value < 0.05 suggests stronger evidence that the covariate has a statistically significant effect on the hazard.

## Results

**Fig. 1:**
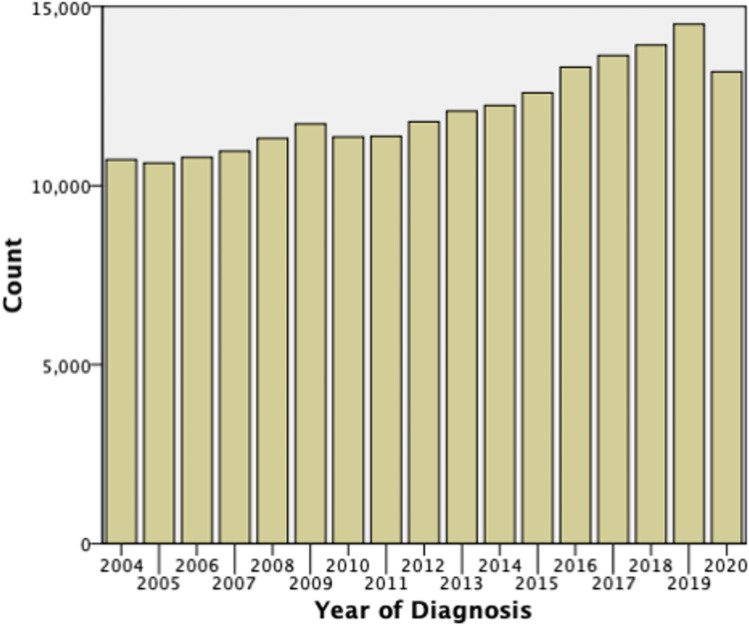
Cases of YBC diagnosed each year. The chart shows the trend that the number of cases diagnosed each year is increasing. There is a drop-off during the year 2020, likely related to the COVID-19 pandemic

Data was obtained from 206,156 of YBC. We see an uptrend in the number of incident cases that are being diagnosed each year. There was a large drop off in the number of incident cases for 2020, which was the year of the COVID-19 pandemic. The number of cases increased from approximately 10,000 in 2004 to almost 15,000 diagnosed in 2019 (Fig. 1).

Overall, there were 55,397 patients diagnosed between 2005 and 2009 (Old) and 67,930 patients diagnosed between 2015 and 2019 (New). Median age in both groups was 37. Approximately 99% of the cases were diagnosed in women (Table 1).

**Table 1:** Comparison of demographic data between Old and New Groups

	1/2005–12/2009	1/2015–12/2019	P value
Total number of patients	N = 55,397	N = 67,930	
Median age	Median = 37 (Range 18–40)	Median = 37 (Range 18–40)	
Sex - Female - Male	N = 55,081(99.4%) N = 316(0.6%)	N = 67,600(99.5%) N = 330(0.5%)	0.045
Race or ethnicity - Non-Hispanic White - Hispanic White - Black - Asian - Pacific Islander - American Indian - Others	N = 34,217(62.9%) N = 4575(8.4%) N = 8695(16.0%) N = 2592(4.8%) N = 90(0.2%) N = 159(0.3%) N = 4092(7.5%)	N = 41,710(62.2%) N = 6720(10.0%) N = 11,156(16.6%) N = 4512(6.7%) N = 255(0.4%) N = 297(0.4%) N = 2431(3.6%)	< 0.001
Insurance - Private - Medicaid - Medicare - Uninsured - Other	N = 43,940(81.7%) N = 5893(11.0%) N = 1023(1.9%) N = 2284(4.2%) N = 659(1.2%)	N = 51,104(76.4%) N = 10,694(16.0%) N = 1434(2.1%) N = 2436(3.6%) N = 1215(1.8%)	< 0.001
Median income - > = $63,000 - Between - < $38,000	N = 20,894(40.2%) N = 23,690(45.6%) N = 7382(14.2%)	N = 23,444(39.7%) N = 27,073(45.8%) N = 8535(14.5%)	0.186
Urban/rural (2013) - Urban - Metro - Rural	N = 4926(9.2%) N = 47,902(89.7%) N = 590(1.1%)	N = 5966(9.0%) N = 59,428(90.0%) N = 657(1.0%)	0.088

Comparison of demographic data between the Old and New group were completed (Table 1). It showed significant increase in the proportion of Hispanic (4575 (8.4%) vs 6720 (10.0%)), Black (8695 (16.0%) vs 11,156 (16.6%)), and Asian (2592 (4.8%) vs 4512 (6.7%)) populations (p < 0.001). There was a significant increase in the proportion of patients with Medicaid (5893 (11.0%) vs 10,694 (16.0%), p < 0.001). There was no significant difference in populations when looking at reported income (p = 0.186). Additionally, there was no significant difference in the type of community patients were living in, with the majority (approximately 90%) of patients reporting living a Metro setting (p = 0.088).

Clinical data was also compared between the Old and New Group (Table 2). For histologic data, there is an increase in the proportion of patients with Invasive Ductal Carcinoma (IDC) (38,282 (69.1%) vs 50,919 (75%)) and Invasive Lobular Carcinoma (ILC) (1357 (2.4%) vs 1823 (2.7%)) with a p value < 0.001. There is also an increase in the proportion of patients whose histology is graded as moderate (17,162 (34.7%) vs 23,631 (38.5%)) with a reduction in the proportion of patients whose histology was graded as poor (26,747 (54.1%) vs 31,732 (51.8%)) with a p value < 0.001. There is a significant increase in the proportion of patients with HR positive disease (HR+) (35,142 (70.0%) vs 49,409 (75.8%), p < 0.001), with a decrease in the proportion of patients who have HR negative disease (HR-) (15,058 (30.0%) vs 15,814 (24.2%), p < 0.001). There is a significant increase in the proportion of patients who did not receive chemotherapy (p < 0.001), although this is likely clinically insignificant given small proportional increase from 29.6 to 30.7%. We see a slight decrease in patients who received radiation therapy from 51.4 to 49.9%, p < 0.01 Unsurprisingly, given the increase in HR + disease, we see a significant increase in the number of patients treated with hormonal therapies (24,447 (46.8%) vs 38,483 (58.3%), p < 0.001).

**Table 2:** Comparison of clinical data between Old and New Groups

	1/2005–12/2009	1/2015–12/2019	P value
Histology - IDC - ILC - DCIS - Other	N = 38,282(69.1%) N = 1357(2.4%) N = 4769(8.6%) N = 10,989(19.8%)	N = 50,919(75.0%) N = 1823(2.7%) N = 5251(7.7%) N = 9,937(14.6%)	< 0.001
Grade - Well - Moderate - Poor - Undiff	N = 4734(9.6%) N = 17,162(34.7%) N = 26,747(54.1%) N = 795(1.6%)	N = 5801(9.5%) N = 23,631(38.5%) N = 31,732(51.8%) N = 153(0.2%)	< 0.001
Clinical T stage - T0 - Tis - T1 - T2 - T3 - T4 - Tx	N = 273(0.6%) N = 6047(12.3%) N = 12,351(25.1%) N = 11,339(23.0%) N = 3519(7.2%) N = 1807(3.7%) N = 13,864(28.2%)	N = 168(0.3%) N = 8952(13.8%) N = 19,940(30.8%) N = 23,837(36.8%) N = 7393(11.4%) N = 2648(4.1%) N = 1813(2.8%)	< 0.001
Clinical N stage - N0 - N1-4 - Nx	N = 24,626(49.9%) N = 10,497(21.3%) N = 14,265(28.9%)	N = 43,548(66.7%) N = 20,711(31.7%) N = 1022(1.6%)	< 0.001
Clinical stage group - 0 - 1 - 2 - 3 - 4	N = 6271(17.8%) N = 9920(28.2%) N = 12,432(35.4%) N = 4450(12.7%) N = 2067(5.9%)	N = 9123(14.6%) N = 19,706(31.5%) N = 21,996(35.2%) N = 7861(12.6%) N = 3784(6.1%)	< 0.001
Subtypes - HR + - HR-	N = 35,142(70.0%) N = 15,058(30.0%)	N = 49,409(75.8%) N = 15,814(24.2%)	< 0.001
Treatment: chemo - Yes - No - Refused	N = 37,133(68.8%) N = 15,973(29.6%) N = 887(1.6%)	N = 45,134(67.1%) N = 20,667(30.7%) N = 1430(2.1%)	< 0.001
Treatment: radiation - Yes - No	N = 27,083(51.4%) N = 25,658(48.6%)	N = 32,874(49.9%) N = 33,041(50.1%)	< 0.001
Treatment: hormonal - Yes - No - Refused	N = 24,447(46.8%) N = 26,511(50.7%) N = 1326(2.5%)	N = 38,483(58.3%) N = 25,195(38.2%) N = 2334(3.5%)	< 0.001

The OS of patients from the New group was compared across various demographic and clinical characteristics (Table 3). Interestingly, race and ethnicity appeared to have a large impact on survival with Black patients having a statistically significant worse overall survival (Hazard ratio 1.24 (1.13–1.35), p < 0.001), while Hispanic White and Asian patients had improved OS (Hazard ratio 0.87 and 0.78, p = 0.024 and p = 0.007, respectively) as compared with Non-Hispanic White patients. Private insurance had the best overall survival, while Medicaid, Medicare, and being uninsured all have worse survival outcomes (p < 0.001). Patients who reported incomes less than $38,000 had worse overall survival as compared to patients who reported to have incomes above $63,000 (p < 0.001). Like historic data, as the stage at diagnosis became more advanced, we saw increasingly worsening OS. The patients who underwent treatment with either chemotherapy, radiation, or hormonal treatment, all had improved OS and those who refused chemotherapy or hormonal treatment having significantly worsened survival (p < 0.001).

**Table 3:** Survival outcomes of New YBC group based on demographic and clinical characteristics

	1/2015–12/2019	Hazard ratio	P value
Total number of patients	N = 67,930		
Median age	Median = 37		
Race Non-Hispanic White Hispanic White Black Asian Pacific Islander American Indian Others Unknown	N = 41,710(61.4%) N = 6720(9.9%) N = 11,156(16.4%) N = 4512(6.6%) N = 255(0.4%) N = 297(0.4%) N = 2431(3.6%) N = 849(1.2%)	1 0.87 (0.77–0.98) 1.24 (1.13–1.35) 0.78 (0.66–0.94) 0.83 (0.47–1.47) 1.38 (0.91–2.09) 0.77 (0.62–0.95)	0.024 < 0.001 0.007 0.524 0.126 0.017
Insurance Private Medicaid Medicare Uninsured Other Unknown	N = 51,104(75.2%) N = 10,694(15.7%) N = 1434(2.1%) N = 2436(3.6%) N = 1215(1.8%) N = 1047(1.5%)	1 1.524317 (1.40146–1.6579) 2.089100 (1.75534–2.4863) 1.560739 (1.33726–1.8216) 0.815201 (0.59073–1.1250)	< 0.001 < 0.001 < 0.001 0.214
Median income > = $63,000 Between < $38,000 Unknown	N = 23,444(34.5%) N = 27,073(39.9%) N = 8535(12.6%) N = 8878(13.1%)	1 1.05 (0.97–1.14) 1.22 (1.09–1.36)	0.196 < 0.001
Urban/rural Urban Metro Rural Unknown	N = 5966(8.8%) N = 59,428(87.5%) N = 657(1.0%) N = 1879(2.8%)	1 0.880206 (0.78241–0.9902) 0.843398 (0.58347–1.2191)	0.034 0.365
Histology IDC ILC DCIS Other	N = 50,919(75.0%) N = 1823(2.7%) N = 5251(7.7%) N = 9937(14.6%)	1 1.072190 (0.83124–1.3830) 0.694982 (0.39966–1.2085) 1.006496 (0.89496 −1.1319)	0.591 0.197 0.914
Grade Well Moderate Poor Undiff Other/unknown	N = 5801(8.5%) N = 23,631(34.8%) N = 31,732(46.7%) N = 153(0.2%) N = 6613(9.7%)	1 1.336603 (1.07333–1.6645) 1.992248 (1.60234–2.4770) 1.714030 (0.86080–3.4130)	0.009 < 0.001 0.125
Clinical T stage T0 Tis T1 T2 T3 T4 Tx Unknown	N = 168(0.2%) N = 8,952(13.2%) N = 19,940(29.4%) N = 23,837(35.1%) N = 7393(10.9%) N = 2,648(3.9%) N = 1813(2.7%) N = 3179(4.7%)	1 0.461401 (0.07266–2.9300) 0.688049 (0.30486–1.5529) 0.690885 (0.30843–1.5476) 0.799413 (0.35625–1.7939) 1.024053 (0.45583 2.3006) 1.234401 (0.52461 2.9045)	0.412 0.368 0.369 0.587 0.954 0.629
Clinical N stage N0 N1-4 Nx Unknown/NA	N = 43,548(64.1%) N = 20,711(30.5%) N = 1022(1.5%) N = 2649(3.9%)	1 1.535898 (1.38942 1.6978) 1.827778 (1.32607 2.5193)	< 0.001 < 0.001
Clinical stage group 0 1 2 3 4 Unknown/NA	N = 9123(13.4%) N = 19,706(29.0%) N = 21,996(32.4%) N = 7861(11.6%) N = 3784(5.6%) N = 5460(8.0%)	1 1.780235 (0.32226–9.8345) 3.511663 (0.63866–19.3087) 6.603445 (1.19827–36.3903) 19.621122(3.57013–107.8361)	0.508 0.149 0.030 < 0.001
Subtypes HR + (ER + or PR +) HR- (ER- and PR-) Unknown/NA	N = 49,409(72.7%) N = 15,814(23.3%) N = 2707(4.0%)	1 1.212311 (1.09181–1.3461)	< 0.001
Treatment: chemo Yes No Refused Unknown	N = 45,134(66.4%) N = 20,667(30.4%) N = 1430(2.1%) N = 699(1.0%)	1 1.217808 (1.06206–1.3964) 2.630723 (2.15541–3.2109)	0.005 < 0.001
Treatment: radiation Yes No Unknown	N = 32,874(48.4%) N = 33,041(48.6%) N = 2015(3.0%)	1 1.206963 (1.11813–1.3028)	< 0.001
Treatment: hormonal Yes No Refused Unknown	N = 38,483(56.7%) N = 25,195(37.1%) N = 2334(3.4%) N = 1918(2.9%)	1 1.996795 (1.79777–2.2179) 2.017254 (1.62610–2.5025)	< 0.001 < 0.001

## Discussion

While the incidence in YBC is increasing, one of the most striking things about the data from the NCDB is the disproportionate effect of this increase on minority populations. When evaluating the race and ethnicity of YBC patients, we see statistically significant increases in the proportion of minority populations, such as Hispanic, Black, and Native American populations, that are being diagnosed with breast cancer. The influence of race and ethnicity becomes even more prominent when observing the variation in OS between racial groups. Most notably, we see that Black patients have a statically significant increased hazard ratio with an overall poorer OS as compared with Non-Hispanic White populations. The poorer OS seen in the Black patients was seen even when controlling for potentially confounding variables such as insurance status, income level, and treatments received. Similar trends have been described in the analysis of cancer mortality within the United States which has found that both Black men and Black women had the highest cancer rate deaths from 2018 to 2020 [19].

The discrepancy is further highlighted when looking at changing socioeconomic status of patients with YBC. One area that can often denote socioeconomic status is the insurance status of patients. Historically, patients with cancer who are uninsured or who have Medicaid as their primary insurance have been shown to present with more advanced disease and typically have a higher mortality [20]. In our study, when comparing the Old and New groups, we see a reduction in the proportion of patients with private insurance. Generally, private insurance tends to be more expensive with greater proportions of patients within higher socioeconomic classes. This contrasts with the increase in patients with Medicaid as their insurance. Medicaid is typically issued to patients who are of lower socioeconomic status, as patients typically qualify based on income status. However, with the expansion of Medicaid that happened through the passing of the Affordable Care Act in 2010, it is possible that this increase is simply a reflection of that expansion given this law was passed between our Old and New study populations. Similar to racial discrepancies, we see within our New group that having Medicaid or being uninsured had a direct negative impact on patient mortality as compared to patients with private insurance. Furthermore, we do see a statistically significant decrease in OS for patients whose median income was less than $38,000. This suggests that YBC patients who are of a lower socioeconomic status generally have worse outcomes.

Overall, this data suggests that there is a disproportionate effect on underserved and underrepresented populations in YBC. Not only have the trends shown that these populations make up a larger proportion of patients diagnosed each year, but also those within these populations who have been diagnosed in recent years have poorer OS. Knowledge of the changing landscape when it comes to YBC is important for clinicians to be aware of and to consider implementation of targeted outreach and support programs for these particular groups.

YBC patients historically have had larger proportion of patients diagnosed with triple negative breast cancer as compared with older patients and often higher grade cancer at diagnosis [21]. However, in our study, we are seeing a significant increase in HR + disease and treatment with hormonal therapies in the New group compared to the Old group. The trend of increasing hormone positivity is seen even when looking at narrower age ranges such as patients 30 years and under or specifically evaluating patients who are 30–40 years old While it is unclear what risk factors may be predisposing young women to develop breast cancer at earlier ages, given the increase in HR + disease, it is possible that there is a significant environmental factor that may be contributing to this increase. Additionally, we do see a survival benefit in patients who have HR + disease and treated with hormonal therapy. However, it is important to consider that these women are often still of childbearing age and treatment with hormonal therapies can potentially have lasting impacts on family planning.

Previous studies have found that the incidence of distant-stage diagnosis of YBC was increasing on average approximately 2% between 1976 and 2009 [22]. When comparing our Old and New group, we do see slight increases in stage 4 breast cancer, however, we see more dramatic increases in earlier stage breast cancer. We see significant increase in N0 staging from approximately 50% to 65% of patients, however, this may be due to the fact that the Old group has significantly more Nx. This may suggest that women are getting diagnosed at earlier stages or could possibly have less aggressive tumors than previous.

While this study does highlight some potentially striking trends in the YBC patient population, it does pose some limitations. The data used was from NCDB which is an already established database and was based on what was reported by numerous medical centers. While this database holds a wealth of information, the questions that we can pose from this data are limited to prior surveys that were previously completed. Also, there are several areas that are reported as “unknown”, which were excluded from the analysis and could skew some of the results of the study. Given the trends we are seeing in this data, further exploration of the factors that are influencing the increasing absolute number of YBC diagnosed each year is warranted.

## Conclusions

Overall, the number of YBC cases are increasing each year. These cases seem to have a disproportionate effect on underserved and underrepresented populations, impacting OS of these patients. There is also an increasing number of HR + cases with treatment with hormonal therapies, which can have large implications on women who are still of childbearing age. Continued research investigating environmental or epigenomic background for the increases in cases, particularly HR + disease, and why they have disproportionate effects on these populations is needed. Given the trends seen in our study, it is important for clinicians to consider targeted outreach efforts and support programs that can help improve outcomes for these underrepresented and underserved populations. Implementation of these targeted programs can help prevent worsening of the divide that appears to be disproportionately affecting these communities.

## Data Availability

No datasets were generated or analysed during the current study.
